# When topicals fall short, oral Janus kinase inhibitors offer a needed solution in chronic hand eczema

**DOI:** 10.1016/j.jdin.2025.06.007

**Published:** 2025-07-17

**Authors:** Christopher G. Bunick

**Affiliations:** a Department of Dermatology, Yale School of Medicine, New Haven, Connecticut; b Program in Translational Biomedicine, Yale School of Medicine, New Haven, Connecticut

**Keywords:** abrocitinib, atopic dermatitis, CHE, delgocitinib, inflammatory skin disease, Janus kinase inhibitors, JAK, ruxolitinibpatient safety, topical therapy, upadacitinib

*To the Editor:* The article by Andersen et al,^1^ “Risk factors that limit use of oral JAK inhibitors in chronic hand eczema: Findings from the Danish Skin Cohort,”^1^ suggests comorbidities may limit oral Janus kinase inhibitors (JAKis) use in chronic hand eczema (CHE). This conclusion warrants scrutiny because (1) the reported 80% prevalence of potentially limiting risk factors lacks a comparator group, and (2) the premise perpetuates the misconception that JAKis are unsafe in patients with common comorbidities, which long-term safety and cardiovascular data in patients with atopic dermatitis (AD) have disproven. Comorbidities like smoking, hypertension, and metabolic syndrome are prevalent in adult dermatology populations. Without appropriate benchmarking, the relative burden in CHE may be overstated.

The authors state, “oral JAKis have black box warnings due to increased risk of cardiovascular events, serious infections, and certain cancers.” This statement is inaccurate and stems from a false premise. The boxed warning in the US Prescribing Information for upadacitinib (UPA) does not state there is “increased risk” of major adverse cardiovascular events, malignancy, or thrombosis. Rather, it notes these events “have occurred” in patients treated with JAKis for inflammatory conditions, citing the tofacitinib oral surveillance trial conducted in older rheumatoid arthritis patients with elevated cardiovascular risk on concomitant methotrexate. These results prompted class-wide labeling as precautionary regulation—not evidence of universal increased risk across all JAKis or indications. Similar boxed warning label language appears for a topical JAKi (ruxolitinib), reinforcing that the warning reflects precaution rather than demonstrated, quantified systemic risk specific to oral JAKis in AD. Boxed warnings guide awareness and clinical judgment, not imply contraindication. Recent data suggest UPA may be cardioprotective in patients with AD, making it unlikely that patients with CHE with similar comorbidities would fare differently on UPA than the population with AD.

Comorbidities alone should not disqualify patients from JAKi therapy. The US Food and Drug Administration and the European Medicines Agency guidance emphasize individualized risk-benefit assessments. In the global UPA AD program, 51% of patients had at least 1 cardiovascular risk factor at baseline. Most patients were treated successfully, with >9000 patient-years of exposure showing very low rates of major adverse cardiovascular events, venous thromboembolism, and malignancy—lower than background rates in real-world moderate-to-severe AD cohorts. These events remained rare among patients with a smoking history or metabolic syndrome, supporting JAK1-selective inhibitor safety and anti-inflammatory function.^2^ Studies show that JAKis do not increase cardiovascular risk.^3^^,^^4^ These data argue that JAKis would be equally safe for patients with CHE who have the same comorbidities.

Oral JAKis, like UPA, demonstrated efficacy in improving CHE among patients with AD, achieving up to a 74% reduction in the hand eczema severity index scores over 16 weeks in randomized trials.^5^ Given frequent overlap between AD and CHE—highlighted by one-third of the CHE cohort having AD history, and patients with AD are 3 to 4 times more likely to develop hand eczema^5^^,^^6^—oral JAKis approved for AD may be an appropriate and evidence-based option for moderate-to-severe or refractory patients with CHE.

Oral JAKis are valuable anti-inflammatory systemic options in CHE, particularly when topicals fail. Treatment decisions should be evidence-based and guided by individual clinical context, disease severity, and therapeutic goals, not by generalized assumptions based on population-level comorbidity rates.

## Conflicts of interest

Dr Bunick has served as an investigator for AbbVie, LEO Pharma, and Timber; a consultant for AbbVie, Incyte, LEO Pharma, Pfizer, Regeneron, and Sanofi.
